# Extensive aortic surgery in acute aortic dissection type A on outcome – insights from 25 years single center experience

**DOI:** 10.1186/s13019-019-1007-7

**Published:** 2019-11-06

**Authors:** Bashar Dib, Philipp Christian Seppelt, Rawa Arif, Alexander Weymann, Gábor Veres, Bastian Schmack, Carsten J. Beller, Arjang Ruhparwar, Matthias Karck, Klaus Kallenbach

**Affiliations:** 1 Department of Cardiac Surgery, University Hospital Heidelberg, Heidelberg, Germany; 2 Department of Medicine, Cardiology, Goethe University Hospital, Frankfurt, Germany; 3 Department of Cardiac Surgery, European Medical School Oldenburg-Groningen, Carl von Ossietzky University Oldenburg, Oldenburg, Germany; 4 Department of Cardiac Surgery, INCCI HaerzZenter, Luxembourg, Luxembourg

**Keywords:** Aortic dissection, Aortic valve sparing, David technique, Aortic arch replacement

## Abstract

**Background:**

This single center study compares the different surgical techniques used in the treatment of acute aortic dissection type A (AADA) analyzing the influence of the extent of the surgical approach on outcome.

**Methods:**

From 1988 to 2012, 407 patients were operated for AADA. The cohort was divided into subgroups according to the surgical approach. These groups were compared with the supracommissural replacement group (SCR; *n* = 141). Groups included aortic valve sparing techniques (AVS; *n* = 29), Composite replacement (COMP; *n* = 119), COMP with total arch replacement (COMP+TAR; *n* = 27) and SCR with TAR (*n* = 75).

**Results:**

Compared to SCR alone, operation (*p* = 0.005), bypass-, cross-clamp and circulatory arrest times were longer in SCR + TAR (all *p* < 0.001). Moreover, operation, bypass and cross clamp times were longer in COMP+TAR (*p* = 0.003, *p* = 0.002 and *p* < 0.001 respectively). COMP alone and AVS required longer cross-clamp time, too (*p* < 0,001 and *p* = 0.002, respectively). Overall 30-day mortality was 21% with the observed lowest rate after AVS (14%, SCR 18%, COMP 25%) but differences in 30-day mortality were not statistically significant. The estimated 10-year survival was 42%, especially AVS demonstrated a good 10-year survival (69%). David technique was superior to Yacoub technique concerning incidence of redo interventions (*p* = 0.036). Risk factors for early mortality included age, circulatory arrest, general malperfusion, bypass and operation time. Circulatory arrest per se was revealed as risk factor for long-term survival.

**Conclusions:**

Within our single center retrospective study concomitant aortic root repair or aortic arch replacement for AADA demonstrated acceptable early and long-term survival. Circulatory arrest, long bypass and operation times per se might be important risk factors for early mortality. AVS techniques can be performed safely and have good outcomes in acute aortic dissection repair.

## Background

Acute aortic dissection type A (AADA) is a life-threatening event that requires emergency surgical management and is associated with high mortality and morbidity [1, 2]. Several surgical approaches have been evolved during the last decades and account for significant outcome improvements [3–5]. Generally, the aortic segments that involve intimal tears should be replaced with a synthetic vascular graft. Supracommissural replacement (SCR) of the ascending aorta remains the most commonly used surgical technique in AADA but leaving parts of the dissected aorta in place. Within the last decades, techniques for complete aortic root replacement were applied and more recently aortic valve-sparing root surgery (for example David and Yacoub technique) was introduced in AADA repair. Furthermore, synthetic vascular grafts with side branches and hybrid prosthesis have been evolved as reliable solutions for the dissected aortic arch and descending aorta. However, despite more sophisticated surgical approaches and continuous improvements in perioperative management, mortality and morbidity in AADA are still high [4, 6]. Aortic root replacement and surgery at the aortic arch and descending aorta require longer operation times including circulatory arrest, all known as important risk factors for short and long-term mortality [4]. This is one important reason why the optimal surgical approach for AADA remains controversial and the discussion about more extensive aortic surgery versus the limited conservative surgery of SCR is still open [4, 7–10]. To address this open question, we report our institutional outcome of the different surgical techniques used in AADA.

## Methods

### Patients characteristics

We operated consecutively 407 patients for AADA from 1988 to 2012 at the Department of Cardiac Surgery of the University Hospital Heidelberg. The patients were treated by different surgeons during that period. The cohort was divided into subgroups according to the surgical approach and all subgroups were compared with the supracommissural replacement group (SCR; *n* = 141; 45%). These groups included aortic valve sparing techniques (AVS; *n* = 29; 7%), Composite replacement (COMP; *n* = 119; 29%), COMP with total arch replacement (COMP+TAR; *n* = 27; 7%) and SCR with TAR (*n* = 75; 18%). Wheat operation (*n* = 7) and AVS with TAR (*n* = 6) were excluded from statistical comparison due to small cohorts. Other nonspecific techniques were used in 3 patients. Seventeen patients (4%, COMP *n* = 2, SCR *n* = 15) underwent Frozen elephant trunk (FET, Jotec® prosthesis, Jotec, Hechingen, Germany) while the classic Elephant-Trunk procedure was accomplished in 16 patients (4%, COMP *n* = 2, SCR *n* = 14). In total, almost a third of the cohort (*n* = 115, 28%) received TAR. Aortic valve-sparing methods were David technique (*n* = 20, 5%) and Yacoub technique (*n* = 15, 4%) and four patients received nonspecific aortic valve reconstruction (1%). Concomitant CABG and concomitant mitral valve operations were performed in 4 patients each (1%).

### Study design

Diagnosis of AADA was confirmed by computed tomography scans, angiography and transthoracic/transesophageal echocardiograms. Mostly, AADA was diagnosed in external centers and patients were transferred to our institution for emergency surgery. If diagnostic studies were incomplete the diagnosis was confirmed in our emergency department. The whole cohort was divided into subgroups according to the surgical approaches. Results of each group were compared to standard SCR group defined as the most conservative but limited surgical approach. Perioperative data, incidence of neurological complications, early mortality and morbidity, cause and risk factors for aortic re-interventions and long-term survival were evaluated. The Ethics Committee of the University of Heidelberg authorized this study (S-286/2010). We have obtained all clinical data retrospectively by reviewing hospital records.

### Definitions

Cardiogenic shock was defined as inadequate tissue perfusion due to reduced cardiac output (cardiac index < 2.2 l/min/m2), manifested low systolic pressure (< 90 mmHg for longer than 30 min). General malperfusion was defined as clinical condition with reduced central and peripheral hypoperfusion caused by longstanding haemodynamic instability causing multiple organ failure. Multi organ failure was assessed by laboratory parameters and clinical signs of organ dysfunction (cold extremities, oliguria or altered mental condition). Acute renal failure was defined as AKIN stage 2 or 3 (AKIN 2: increase of serum creatinine > 200 a 300% (> 2 a3x) or < 0.5 mL/kg/h in 12 h) according to the Acute Kidney Injury Network (AKIN) classification. Stroke was specified as the presence of new neurological symptoms or was verified by computed tomography scan or magnetic resonance imaging of the head.

### Follow-up

Follow-up data was obtained from the patient directly, or by contacting the local population administration office, home doctors or patient’s family. Completeness of follow-up was 97% with a mean follow-up time of 4.5 ± 5.6 years (up to 25 years).

### Surgical technique

At the first years of the study period, femoral arterial and venous cannulation was the most often used approach for establishment of extracorporeal circulation. With beginning of the second millennium, standard surgical approach was median sternotomy with direct arterial cannulation of the ascending aorta and venous cannulation by a two-stage right-atrial cannula. In case of hemodynamic instability or suspected large thoracic aneurysm, the femoral artery and femoral vein were further used for cannulation and initiation of extracorporeal circulation (ECC). In more recent times, axillary arterial cannulation was applied most often as the standard cannulation technique. After systemic administration of heparin, ECC was established (targeted activated clotting time > 400 s). For decompression of the left ventricle, a vent catheter was inserted via the upper right pulmonary vein. After initiation of ECC, the patient was cooled to target body temperature. After aorta was cross-clamped and ascending aorta was transected above the commissures, cardioplegic solution was administrated selectively in antegrade fashion or retrograde perfusion via the coronary sinus was implemented. The decision for the surgical approach was taken individually depending on the clinical status of the patient and on the intraoperative findings, which included the location of the intimal tear and the inspection of aortic root and aortic valve. The surgical decision (e.g. for aortic arch replacement) depended also on the existence of a patent intimal entry or reentry within the aortic arch. We aimed to resect all aortic segments that contained an intimal tear and to restore the native aortic circulation. Surgical techniques varied from simple SCR to a complete replacement of the aortic root or a complete aortic arch replacement with or without applying a hybrid frozen elephant procedure as surgical solution for an affected descending thoracic aorta.

We used Gelatine-resorcinol-formaldehyde (GRF) glue to readapt the dissected distal aortic wall layers. Deep systemic hypothermic circulatory arrest was also applied when necessary. In the more recent cases, moderate hypothermic circulatory arrest with antegrade cerebral perfusion was used during the inspection of the aortic arch or, if required, during replacement of the aortic arch. Cerebral oximetry (INVOS™, Medtronic, Dublin, Ireland) and in the early years common electroencephalography were used routinely to monitor cerebral blood oxygen saturation during or electrical brain activity during AADA repair. In the case of circulatory arrest, once distal aortic anastomosis was accomplished, the Dacron prosthesis was cannulated, clamped proximally and full reperfusion and rewarming was started. During active rewarming, the proximal anastomosis was completed and concomitant procedures could be performed. After completion of the proximal anastomosis the lungs were manually re-inflated and the de-airing was performed before aortic cross-clamp was released. Atrial and ventricular pacemaker electrodes were placed to ensure a heart rate between 70 and 100 beats/min. Once the patient was rewarmed to a body core temperature above 36 °C and after sufficient reperfusion time and generation of optimal preload, weaning from ECC was performed in the usual manner. Thorax drainages were placed and the chest was closed after meticulous hemostasis was accomplished.

### Statistical analysis

For data description and analysis, SPSS statistic software was used (IBM®SPSS®22, 2013, Chicago, USA). Variables are described as quantity and percentages or as means and standard deviations. Statistical analysis was performed using Student’s T-test, if variances were not equal (tested by Leven’s test) Mann-Whitney-U-Test was performed*.* Chi-squared test (Fisher exact tests if *n* ≤ 5) was used for categorical variables. To determine perioperative risk factors for early mortality and re-operations, logistic regression testing were performed. The impact of perioperative variables on long-term survival was analyzed by multivariable cox proportional hazards model. For adjusting the logistic and cox proportional hazards model to relevant baseline parameters, the variables age, sex and the individual operation methods (SCR, SCR + TAR, COMP, COMP + TAR, AVS) were included into the multivariate model. First, possible relevant risk factors were tested with this adjusted model by backward LR stepwise selection (LR, Likelihood Ratio, selection and significance criteria *p* < 0.1 and *p* < 0.05 respectively). Final models were calculated by entering all selected variables *en bloc* in a single step (Enter mode). Kaplan–Meier analysis was used to estimate survival. Two-tailed significance level was determined 5%.

## Results

### Preoperative presentations and patient characteristics

The overall cohort included 132 (32%) female and 285 male patients with a mean age of 58 years (Table 1). Thirty percent (*n* = 123) were type 2 dissection according to DeBakey classification (DeBakey type 3 were not included). Male gender was clinically more frequent in COMP groups (*n* = 89, 75% in COMP and *n* = 21, 78% in COMP+TAR. Relevant secondary diagnoses were bicuspid aortic valve (*n* = 21, 5.2%) and Marfan syndrome (*n* = 15, 3.7%). Twelve AADA cases (*n* = 3% = were caused iatrogenically, 12 occurred after trauma (*n* = 3%) and eight during pregnancy (2%). Forty-nine patients (12%) had a history of cardiothoracic surgery and 42 had already experienced myocardial infarction at the time of presentation (10.3%). Sixty-three patients (16%) presented in cardiogenic shock or had required cardiopulmonary resuscitation (*n* = 22, 5.4%). As clinical correlate for bad medical condition patients suffered general malperfusion (*n* = 80, 20%), coronary malperfusion (*n* = 42, 10.3%) or showed new neurological symptoms (*n* = 57, 14%). In comparison with SCR patients were younger in COMP (61y vs. 56y, *p* = 0.017) and COMP+TAR (61y vs. 51y, *p* = 0.001) group. Proportion of male patients was higher in AVS than in SCR-group (*n* = 24, 82% vs. *n* = 87, 61%, *p* = 0.025). Additionally, patients of COMP group presented more frequently in cardiogenic shock (*n* = 18, 13% vs. *n* = 27, 23%, *p* = 0.032, Table 1).

**Table 1:** Patients characteristics

	All	SCR	SCR + TAR	*p*-value	COMP	*p*-value	COMP+TAR	*p*-value	AVS	*p*-value
Cohort number	407	141 (35)	75 (18)	–	119 (29)	–	27 (7)	–	29 (7)	–
Gender, male	285 (70)	87 (61)	52 (69)	0.224	89 (75)	0.018	21 (78)	0.097	24 (82)	0.025
Age (years)	58 ± 13	61 ± 13	58 ± 11	0.162	56 ± 14	0.017	51 ± 13	0.001	58 ± 11	0.304
Marfan syndrome	15 (3.7)	4 (3)	4 (5)	0.417	5 (4)	0.587	1 (4)	0.816	0 (0)	0.345
DeBakey type 2^a^	123 (30)	49 (35)	0 (0)	< 0.001	50 (42)	0.160	13 (48)	0.520	9 (31)	0.927
Bicuspid aortic valve	21 (5.2)	5 (4)	5 (7)	0.336	3 (3)	0.603	3 (12)	0.089	3 (11)	0.130
Previous surgery	49 (12)	20 (14)	9 (12)	0.568	16 (14)	0.728	1 (4)	0.160	1 (3)	0.088
Pericardial effusion	120 (30)	38 (27)	25 (33)	0.342	34 (29)	0.766	6 (22)	0.067	12 (41)	0.126
Resuscitation	22 (5.4)	8 (6)	0 (0)	0.036	10 (8)	0.388	2 (11)	0.067	1 (4)	0.626
Shock	63 (16)	18 (13)	7 (9)	0.301	27 (23)	0. 032	4 (15)	0.068	5 (18)	0.473
General malperfusion	80 (20)	35 (26)	10 (14)	0.042	24 (21)	0.448	2 (8)	0.065	7 (25)	0.950
Coronary malperfusion	42 (10.3)	19 (14)	5 (7)	0.101	11 (10)	0.267	2 (8)	0.424	2 (7)	0.302
New neurological symptoms	57 (14)	17 (12)	15 (20)	0.123	12 (10)	0.601	8 (31)	0.003	2 (7)	0.416

Data are presented as n (percentage) or mean ± standard deviation (SD). All groups were compared to standard SCR

*AADA* acute aortic dissection type A, *AVS* aortic valve sparing, *COMP* composite replacement, *SCR* supracommissural replacement, *TAR* total arch replacement

^a^Since Debakey type 3 dissection were not included, rest of the cohort were DeBakey type 1 dissections

### Intraoperative findings and results

Peripheral cannulation of the femoral vessels for initiation of extra corporal circulation (ECC) was carried out in 209 patients (52%) while direct aortic cannulation was performed in 170 patients (42%) and subclavian artery or axillary artery cannulation was performed in 26 patients (6%). Overall, mean bypass time was 221 min with a mean cross-clamp time of 115 min (Table 2). Selective cerebral perfusion technique was used in 136 patients (33%). Operative procedures took longer if TAR was accomplished (SCR + TAR 337 min vs. 387 min, *p* = 0.005 and COMP+TAR 337 min vs. 420 min, *p* = 0.003 compared to SCR). ECC and cross-clamp times were longer in SCR + TAR (199 min vs. 255 min, *p* < 0.001 and 95 min vs. 123 min, *p* < 0.001 respectively) and COMP+TAR (199 min vs. 263 min, *p* = 0.002 and 95 min vs. 137 min *p* < 0.001 respectively) than in SCR. However, circulatory arrest time was only significantly longer in SCR + TAR (22 min vs. 42 min, *p* < 0.001, compared to SCR). Circulatory arrest times were the shortest in COMP and AVS operations (16mins and 15 min respectively). If COMP or AVS were performed, such as David or Yacoub operation, longer aortic cross-clamp times (95 min vs. 124 min, *p* < 0.001 and 95 min vs. 122 min, *p* = 0.002, respectively) but comparable ECC-times were required compared to SCR alone (199 min vs. 218 min, *p* = 0.068 and 199 min vs. 202 min, *p* = 0.860, respectively). Overall, intraoperative mortality was 7.3% (*n* = 29) with the highest mortality in COMP+TAR group (*n* = 4, 15%, *p* = 0.018 compared to SCR).

**Table 2:** Intraoperative findings and early postoperative outcome

	all	SCR	SCR + TAR	*p*-value	COMP	*p*-value	COMP+TAR	*p*-value	AVS	*p*-value
Operation time (min)	359 ± 142	337 ± 123	387 ± 147	0.005	362 ± 159	0.118	420 ± 164	0.003	326 ± 128	0. 728
ECC time (min)	221 ± 96	199 ± 90	255 ± 115	< 0.001	218 ± 85	0.068	263 ± 110	0.002	202 ± 85	0. 860
Cross-clamp time (min)	115 ± 52	95 ± 42	123 ± 67	< 0.001	124 ± 47	< 0.001	137 ± 55	< 0.001	122 ± 46	0.002
Circulatory arrest time (min)	25 ± 28	22 ± 27	42 ± 34	< 0.001	16 ± 19	0.047	29 ± 29	0.256	15 ± 15	0.144
30-day mortality	83 (21)	25 (18)	14 (19)	0.888	29 (25)	0.202	6 (24)	0.513	4 (14)	0.556
Intraoperative mortality	29 (7.3)	5 (4)	5 (7)	0.304	11 (9)	0.056	4 (15)	0.018	1 (3)	0.974
Reexploration for bleeding	38 (9.3)	9 (7)	1 (11)	0.189	12 (10)	0.201	2 (8)	0.716	8 (29)	< 0.001
New stroke	52 (12)	15 (11)	12 (18)	0.493	21 (19)	0.270	5 (22)	0.337	1 (14)	0.963
ICU stay (days)^a^	4 (1–9)	4 (1–4)	5 (1–5)	0.753	3 (1–6.5)	0.036	4 (1–8.5)	0.691	3 (1–8)	0.397
Mechanical ventilation (h)	98 ± 208	121 ± 286	91 ± 145	0.474	75 ± 137	0.162	65 ± 188	0.279	88 ± 154	0.613
Hemodialysis	57 (14)	19 (15)	15 (22)	0.179	16 (15)	0.819	4 (17)	0.688	2 (7)	0.315
Blood transfusion (ml)	2111 ± 1534	2422 ± 3938	2203 ± 2115	0.894	2230 ± 3034	0.935	4186 ± 4929	0.142	2842 ± 4258	0.492

Data are presented as n (percentage) or mean ± standard deviation (SD)

All groups were compared to standard SCR. *AVS* aortic valve sparing, *COMP* composite replacement, *ECC* extracorporeal circulation, *ICU* intensive care unit, *SCR* supracommissural replacement, *TAR* total arch replacement

^a^median (interquartile range, Mann-Whitney-U Test)

### Postoperative early outcome

**Fig. 1:**
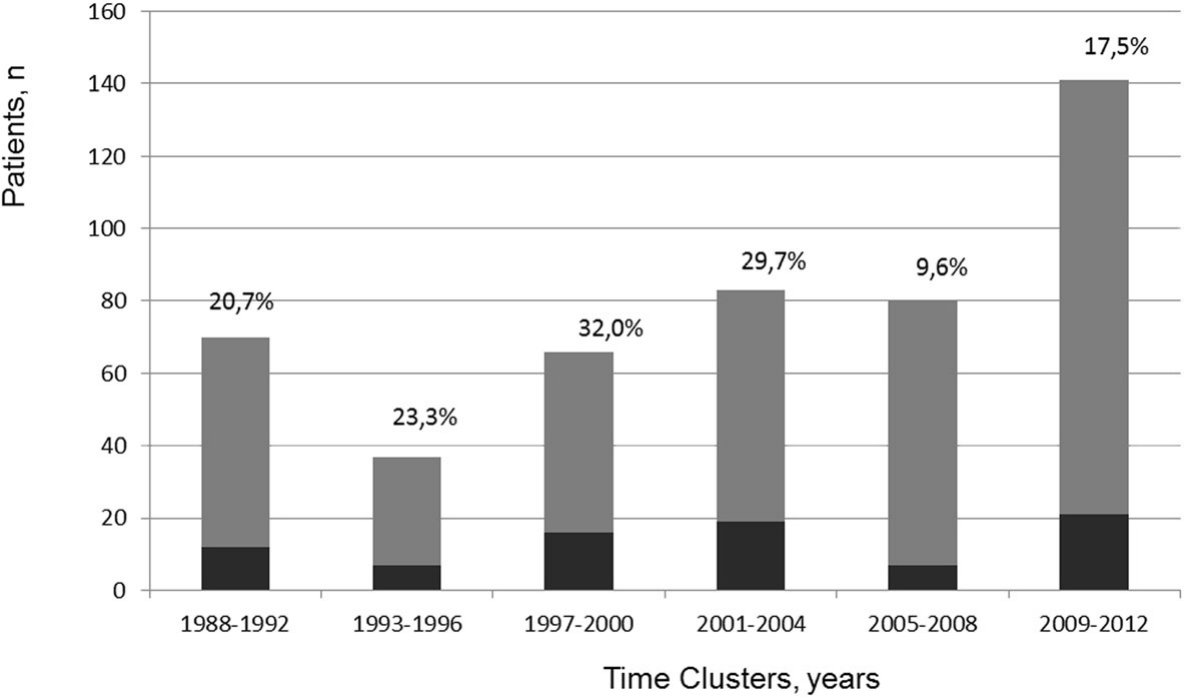
Number of patients operated on AADA in our institution and percentage of early mortality over the course of years, subdivided in year-clusters

The median stay on intensive care unit was 4 days (interquartile range 1–9 days) with a mean mechanical ventilation time of 91 h (standard deviation ±208 h) (Table 2). Stay on intensive care unit was shorter after COMP compared to SCR (median 4d (1–4) vs 3d (1–6.5), *p* = 0.036). Sixty-nine patients (17%) developed renal failure postoperatively and 57 (14%) required temporary hemodialysis. Postoperative neurological complications such as stroke (not reported preoperatively) occurred in 52 (12%) cases. Patients undergoing AVS required significant more re-explorations with re-thoracotomy due to postoperative bleeding compared to patients receiving SCR (*n* = 9, 7% vs. *n* = 8, 29%, *p* < 0.001). Our cohort had an overall 30-day mortality of 21% without statistically significant differences between surgical subgroups. However, 30-day mortality was observed markedly higher after COMP than after AVS (*n* = 29, 25% vs. *n* = 4, 14%, *p* = 0.32). By analyzing the number of operated patients over the course of years we observed an increase of operations for AADA per year and improvement of early mortality during the last 8 years (Fig. 1).

### Results of long-term follow-up

**Fig. 2:**
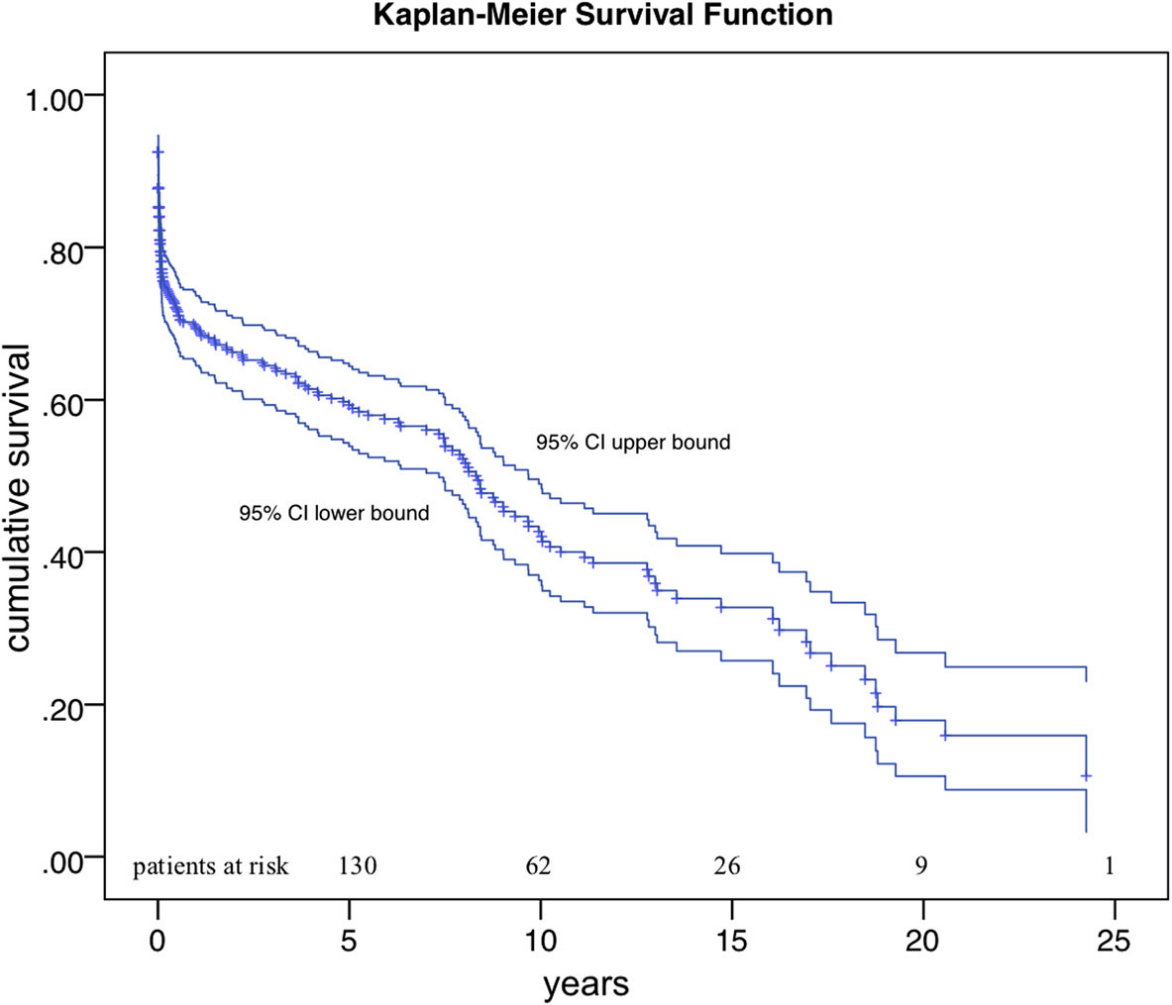
Estimated overall survival (Kaplan Meier survival function) of the cohort and patients at risk with 95% confidential interval boundaries

**Fig. 3:**
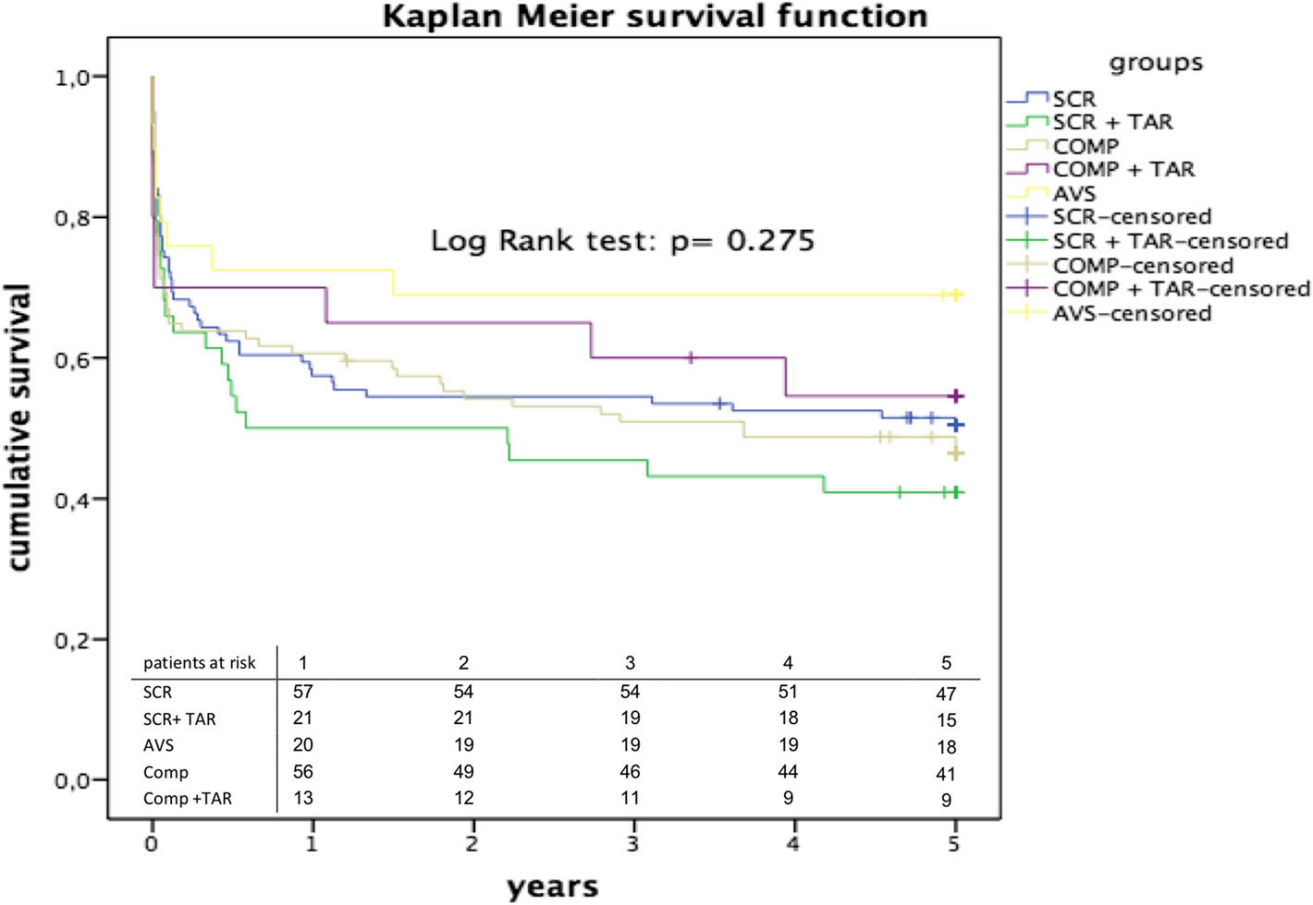
Kaplan Meier survival function with estimated 5-year survival after aortic valve sparing (AVS), composite replacement (COMP), supracommissural replacement (SCR) and total arch replacement (TAR)

Kaplan-Meier survival curve analysis revealed an estimated-overall survival for 1, 5 and 10 years of 69, 58.9 and 42% respectively (Fig. 2 and Table 3). The 5-years survival estimated by Kaplan Meier function was the highest after AVS (69.9%) and the lowest after SCR + TAR (46.1%, Table 3, Fig. 3). In the overall cohort, freedom from re-operation was 92% over the total follow-up, with an estimated 5-year survival of 78% after redo operation (64.4–94.2, 95% CI).

**Table 3:** Short and long-term survival

Group	30d	1y	5y	10y	Median survival, years
Overall	78.2 (82.2–74.1)	69 (64.4–73.6)	58.9 (53.6–64.2)	42 (35.6–48.4)	8.4 (7.3–9.5)
SCR	77.8 (70.7–85)	64.5 (56.3–72.7)	57.3 (48.3–66.2)	34.3 (23.3–45.3)	8.1 (5.5–10.8)
SCR + TAR	75 (66.2–86.9)	63.5 (51.5–75.5)	46.1 (31.2–61-1)	–	4.2 (0.5–7.9)
COMP	70.1 (61.4–79)	64.2 (55.1–73)	53.7 (43.7–63-6)	39.9 (29.2.50.7)	7.9 (2.5–13.4)
COMP + TAR	70.9 (52.6–89.2)	70.9 (52.6–89.2)	46.8 (24–69.7)	35.1 (8.9–61.4)	7.5 (0.0–19.6)
AVS	80.6 (67.6–93.5)	74.6 (60.2–89)	69.6 (53.2–86)	69.6 (29.3–76.8)	13.1 (7.4–18.7)

Estimated short and long-term survival generated by Kaplan Meier Analysis. Data is shown in percentage, % (95% CI)

*AVS* aortic valve sparing, *CI* confidence interval, *COMP* composite replacement, *d* days, *SCR* supracoronar replacement, + *TAR* total arch replacement, *y* years

### Risk factors for early and late outcome

Multivariable logistic regression revealed long circulatory arrest time as strong independent risk factor for early mortality (Odds Ratio = 13, death within 30 days post-surgery). Other variables were also identified as independent factors influencing the early mortality, such as general malperfusion and bypass- and operation times (Table 4).

**Table 4:** Multivariable logistic regression for risk factor analysis of 30-day mortality

Variable	*p*-value	OR (95% CI)
SCR^a^	.229	.327 (.05–2.02)
SCR + TAR^a^	.474	.712 (.28–1.80)
COMP^a^	.713	.919 (.59–1.44)
COMP+TAR^a^	.475	.842 (.53–1.35)
AVS^a^	.687	.877 (.46–1.66)
Sex^a^	.518	.773 (.35–1.69)
Age^a^	.109	1.024 (.99–1.05)
Circulatory arrest	.000	13.098 (3.78–45.36)
Bypass time	.001	1.014 (1.01–1.02)
General malperfusion	.001	3.411 (1.61–7.22)
Operation time	.033	.995 (.99–1.00)
Hemodialyses	.175	1.839 (.76–4.43)
Myocardial infarction	.192	2.330 (.66–8.31)
aortic cross clamp times	.329	.995 (.98–1.01)
Sepsis	.521	1.489 (.44–5.02)
Hypertonia	.660	1.180 (.57–2.46)

*AVS* aortic valve sparing, *CI* confidence interval, *COMP* composite replacement, *OR* odds ratio, *SCR* supracommissural replacement, *TAR* total arch replacement

^a^Variables were fixed in the model for adjustment. General malperfusion, sepsis, hypertonia and myocardial infarction diagnosed prior initial surgery; hemodialysis required postoperatively

Numerous variables impacting the overall survival were found by Cox regression. Multivariable analysis confirmed age, circulatory arrest, preoperative general malperfusion, postoperative need for hemodialysis and bypass time as independent factors influencing the overall survival (Table 5). Subanalysis identified younger age (*p* = 0.003, HR (hazzard ratio) 0.95; 0.92–0.98, 95% CI) and Marfan syndrome (*p* = 0.001; OR 8.72, 2.40–31.6 95%) as independent risk factors for root re-operation (multivariable logistic regression analysis, not shown).

**Table 5:** Multivariable cox proportional hazard regression model for risk factor analysis of long-term survival

Variable	*p*-value	HR
SCR^a^	.182	.509 (.19–1.37)
SCR + TAR^a^	.451	.823 (.496–1.37)
AVS^a^	.110	.739 (.51–1.07)
COMP^a^	.152	.835 (.65–1.07)
COMP+TAR^a^	.248	.860 (.67–1.11
Sex^a^	.683	.928 (.65–1.33)
Age^a^	.000	1.028 (1.01–1.04)
Circulatory arrest	.000	2.891 (1.60–5.2)
Bypass time	.001	1.007 (1.00–1.01
Operation time	.019	.997 (.99–1.00)
General malperfusion	.029	1.530 (1.05–2.24)
Hemodialyses	.031	1.701 (1.05–2.76)
Aortic cross clamp times	.141	.996 (.99–1.00)
Previous cardiothoracic surgery	.160	1.410 (.87–2.28)
Myocardial infarction	.263	1.526 (.73–3.2)
Sepsis	.657	1.164 (.60–2.27)

*AVS* aortic valve sparing, *CI* confidence interval, *COMP* composite replacement, *HR* hazzard ratio, *SCR* supracommissural replacement, *TAR* total arch replacement

^a^Variables were fixed in the model for adjustment. General malperfusion, sepsis and myocardial infarction diagnosed prior initial surgery; hemodialysis required postoperatively

## Discussion

Primary goal of surgery for AADA, a life-threatening event, remains to save the patient’s life. Today, surgical approaches for AADA repair range from simple SCR to complete thoracic aortic replacement with aortic valve reconstruction. Surgery in AADA should not only treat acute complications but should also prevent or at least minimize the risk for early and late complications after aortic dissection (e.g. downstream aneurysm formation) [11]. This approach demands a repair of all dissected parts of the aorta, in most cases including the aortic arch and descending aorta. However, extensive surgical procedures for AADA, such as aortic root replacement, TAR or hybrid procedures for concomitant dissection of the downstream aorta, remain controversial [3, 4, 6, 12]. Furthermore, although the surgical outcomes of AADA have been improved significantly during the last decades, standardization in repair techniques is still lacking [3–5]. Moreover, the surgical strategy usually still depends on a surgeon’s preference and experience.

In the first years of the study period, femoral artery cannulation was the widest used cannulation technique. However, direct aortic cannulation was later on preferred by several surgeons who joined the team in the 2000 years. Direct aortic cannulation (preferably with use of Seldinger technique) can be performed in experienced hands with good results, as proofed by several publications [13]. However, during the last years of the study period and with growing evidence that axillary cannulation is superior to direct aortic cannulation, axillary cannulation became standard cannulation technique [14].

SCR is technically the simplest approach, the fastest surgical strategy and the most common used method for AADA repair. Many studies reported lower perioperative mortality after limited surgical strategy (such as simple SCR technique) but on the other hand higher rates of late complications with need for re-interventions [7, 10, 15, 16]. On the contrary, more extended surgery, such as aortic root replacement and TAR with or without stenting of the descending aorta (frozen elephant trunk technique), has proved to reduce late dissection complications and the need for re-interventions [11, 17, 18]. Contradictory data in literature is the reason why the best surgical approach still remains uncertain.

Although patients were younger in the concomitant aortic root and arch replacement group (COMP+TAR), intraoperative mortality was observed higher. This finding may be explained by a combination of longer operation and circulatory arrest times. Early mortality comparisons, however, were statistically insignificant.

Our study results stand in contrast to several previous reports indicating that favorable outcome is reduced if aortic arch surgery was undertaken [10, 19–22]. This report revealed comparable long-term survival rates for SCR and operations with extended aortic arch repair. Our results are supported by GERAADA (German Registry for Acute Aortic Dissection Type A) results and many other recent studies [4, 18, 23]. Therefore, we suppose that more extensive aortic arch surgery does not cause worse overall outcome in principle.

Based on GERAADA registry data, containing 2137 surgically treated patients with AADA between year 2006 and 2010, Conzelmann et al. reported an early mortality of 16.9% in patients with AADA [4]. Within our cohort the overall early mortality was relatively high (21%). The long retrospective study period in our cohort (back to year 1988) may explain our overall inferior results. During more recent time periods, our results are comparable to the reported mortality in GERAADA. Besides new and more sophisticated surgical strategies, the general improvements in perioperative treatment over the last decades may have impacted the overall outcome for patients with AADA. Our relatively poor early survival rate is supported by the findings of similar reports with comparable long retrospective study periods [5, 6, 24].

As it has been reported previously, we did not detect superiority of a specific surgical technique concerning long-term survival [4, 23]. However, our patients from the AVS group showed favorable early and long-term outcomes but with only a trend towards survival improvement. Many factors may explain these good results of AVS in patients with AADA: First, surgeons operating AVS usually have more surgical experience. Second, the majority of these AVS operations had been performed more recently (after year 2006). Third, AVS technique is usually not considered in high-risk patients and advanced aortic disease. On the other hand, we have to underline, that our results also indicate an increased risk for postoperative bleeding with need for re-thoracotomy after AVS (29%). Interestingly this finding seems not to have any impact on short- and long-term survival in our cohort. Even if the scientific evidence is still weak, in our opinion AVS should always, as long as technically feasible, be the first choice for experienced surgeons if the aortic root is affected. Especially young patients (with or without Marfan syndrome) benefit from AVS, because a lifetime oral anticoagulation is not required.

Need for re-operation is an important factor impacting the quality of life of patients with AADA. Within our cohort younger age and Marfan syndrome were identified as risk factors for redo operation. The estimated 5-year survival within our cohort was 78% after redo operation and is comparable to the results of recent publications [25, 26]. The question for the best AVS technique in AADA, Yacoub or David technique, has not been answered yet. Theoretically, and based on our experience the David procedure requires longer operation times. A significant difference in short and long-term survival was not observed. But in our relatively small cohort of AADA patients receiving AVS (*n* = 35), the observed need for re-aortic root operations was less frequent after David technique compared to Yacoub. Because David is the more recent and advanced technique and on the other hand Yacoub technique has been performed at earlier study period at our institution, time is about to tell if David operation is really the favorable AVS approach. In the literature, there is a trend towards better valve longevity for patients after the David procedure compared to the Yacoub technique [27, 28].

Similar to the results of the registry data of GERAADA, our findings indicate the predictive value of neurological complications, circulatory arrest, bypass and operation time as important risk factors for early mortality [(4)]. Beyond that and as previously reported by Goda et al., freedom from cardiac circulatory arrest and preoperative cerebral malperfusion are valid predictors for long-term survival [23]. The duration of circulatory arrest seems to have a strong impact on general outcomes. As mentioned, patients with AADA and affected aortic arch may benefit from extended aortic arch surgery in circulatory arrest but in general longer circulatory arrest time seems to limit the overall outcome. The upcoming results of the big registry data such as GERAADA will have to tell us, how this small path between extended aortic arch surgery and limitation of circulatory arrest time has to look like finally.

### Limitations

Our single-center study has an exploratory character and based exclusively on retrospective analysis of surgical strategies used in AADA repair. An important limitation is the long study period over 25 years that included the change and development of surgical techniques during the study period. Moreover, our patient cohort is inhomogeneous due to the natural individual characteristic of AADA. Furthermore, surgical technique was chosen individually according to clinical presentation and, more dominantly, according to surgeon’s preference. The influence impact of the surgeon per se was not measured.

## Conclusions

Within our retrospective single center study concomitant aortic arch or aortic root surgery in AADA demonstrated good long-term outcomes even though circulatory arrest, long bypass and operations times per se were revealed as important risk factors for early mortality. The fastest surgical strategy might not be necessarily the best option. AVS techniques can be performed safely and have good outcomes in patients with AADA and limited aortic disease despite longer operation times. Our results indicate that the experienced surgeon has not to fear complex and time-consuming aortic arch surgery for AADA repair but should always adapt his surgical strategy for each individual patient.

## Data Availability

The datasets used and/or analysed during the current study are available from the corresponding author on reasonable request.

## Abbreviations

AADA: Acute aortic dissection type A
AKIN: Acute Kidney Injury Network
AVS: Aortic valve sparing
COMP: Composite replacement
ECC: Extracorporeal circulation
FET: Frozen elephant trunk
GERAADA: German Registry for Acute Aortic Dissection Type A
GRF: Gelatine-resorcinol-formaldehyde
HR: Hazzard ratio
ICU: Intensive care unit
NYHA: New York Heart Association
OR: Odds ratio
SCR: Supracommissural replacement
TAR: Total arch replacement

## References

[1] Hagan PG, Nienaber CA, Isselbacher EM, Bruckman D, Karavite DJ (2000). The international registry of acute aortic dissection (IRAD): new insights into an old disease. JAMA.

[2] Erbel R, Aboyans V, Boileau C, Bossone E, Bartolomeo RD (2014). 2014 ESC guidelines on the diagnosis and treatment of aortic diseases. Document covering acute and chronic aortic diseases of the thoracic and abdominal aorta of the adult The Task Force for the Diagnosis and Treatment of Aortic Diseases of the European Society of Cardiology (ESC). Eur Heart J.

[3] Kallenbach K, Oelze T, Salcher R, Hagl C, Karck M (2004). Evolving strategies for treatment of acute aortic dissection type a. Circulation.

[4] Conzelmann LO, Weigang E, Mehlhorn U, Abugameh A, Hoffmann I (2016). Mortality in patients with acute aortic dissection type a: analysis of pre- and intraoperative risk factors from the German registry for acute aortic dissection type a (GERAADA). Eur J Cardiothorac Surg.

[5] Knipp BS, Deeb GM, Prager RL, Williams CY, Upchurch GR, Patel HJ (2007). A contemporary analysis of outcomes for operative repair of type a aortic dissection in the United States. Surgery.

[6] Berretta P, Patel HJ, Gleason TG, Sundt TM, Myrmel T (2016). IRAD experience on surgical type a acute dissection patients: results and predictors of mortality. Ann Cardiothorac Surg.

[7] Kim JB, Chung CH, Moon DH, Ha GJ, Lee TY (2011). Total arch repair versus hemiarch repair in the management of acute DeBakey type I aortic dissection. Eur J Cardiothorac Surg.

[8] Kazui T, Yamashita K, Washiyama N, Terada H, Bashar AH (2002). Impact of an aggressive surgical approach on surgical outcome in type a aortic dissection. Ann Thorac Surg.

[9] Tan ME, Dossche KM, Morshuis WJ, Kelder JC, Waanders FG, Schepens MA (2003). Is extended arch replacement for acute type a aortic dissection an additional risk factor for mortality?. Ann Thorac Surg.

[10] Rylski B, Beyersdorf F, Kari FA, Schlosser J, Blanke P, Siepe M (2014). Acute type a aortic dissection extending beyond ascending aorta: limited or extensive distal repair. J Thorac Cardiov Sur.

[11] Shrestha M, Bachet J, Bavaria J, Carrel TP, De Paulis R (2015). Current status and recommendations for use of the frozen elephant trunk technique: a position paper by the vascular domain of EACTS. Eur J Cardiothorac Surg.

[12] Moon MR, Sundt TM, Pasque MK, Barner HB, Huddleston CB (2001). Does the extent of proximal or distal resection influence outcome for type a dissections?. Ann Thorac Surg.

[13] Kamiya H, Kallenbach K, Halmer D, Ozsoz M, Ilg K (2009). Comparison of ascending aorta versus femoral artery cannulation for acute aortic dissection type a. Circulation.

[14] Tiwari KK, Murzi M, Bevilacqua S, Glauber M (2010). Which cannulation (ascending aortic cannulation or peripheral arterial cannulation) is better for acute type a aortic dissection surgery?. Interact Cardiovasc Thorac Surg.

[15] Cho K, Jeong J, Park J, Yun S, Woo J (2016). Long-term changes in the distal aorta after aortic arch replacement in acute DeBakey type I aortic dissection. Korean J Thorac Cardiovasc Surg.

[16] David TE, Armstrong S, Ivanov J, Feindel CM, Omran A, Webb G. Results of aortic valve-sparing operations. 2001;122:39–46. 10.1067/mtc.2001.112935 11436035

[17] Omura A, Miyahara S, Yamanaka K, Sakamoto T, Matsumori M (2016). Early and late outcomes of repaired acute DeBakey type I aortic dissection after graft replacement. J Thorac Cardiovas Sur.

[18] Rice RD, Sandhu HK, Leake SS, Afifi RO, Azizzadeh A (2015). Is Total arch replacement associated with worse outcomes during repair of acute type a aortic dissection?. Ann Thorac Surg.

[19] Crawford ES, Kirklin JW, Naftel DC, Svensson LG, Coselli JS, Safi HJ (1992). Surgery for acute dissection of ascending aorta. Should the arch be included?. J Thorac Cardiovas Sur.

[20] Borst HG, Buhner B, Jurmann M (1994). Tactics and techniques of aortic arch replacement. J Card Surg.

[21] Miller DC, Mitchell RS, Oyer PE, Stinson EB, Jamieson SW, Shumway NE (1984). Independent determinants of operative mortality for patients with aortic dissections. Circulation.

[22] Poon SS, Theologou T, Harrington D, Kuduvalli M, Oo A, Field M (2016). Hemiarch versus total aortic arch replacement in acute type a dissection: a systematic review and meta-analysis. Ann Thorac Surg.

[23] Goda M, Imoto K, Suzuki S, Uchida K, Yanagi H (2010). Risk analysis for hospital mortality in patients with acute type a aortic dissection. Ann Thorac Surg.

[24] Tan ME, Dossche KM, Morshuis WJ, Knaepen PJ, Defauw JJ (2003). Operative risk factors of type a aortic dissection: analysis of 252 consecutive patients. Cardiovasc Surg.

[25] Rahnavardi M, Yan TD, Bannon PG, Wilson MK (2011). Aortic valve-sparing operations in aortic root aneurysms: remodeling or reimplantation?. Interact Cardiovasc Thorac Surg.

[26] Colli A, Carrozzini M, Galuppo M, Comisso M, Toto F (2016). Analysis of early and long-term outcomes of acute type a aortic dissection according to the new international aortic arch surgery study group recommendations. Heart Vessels.

[27] Kari FA, Doll KN, Hemmer W, Liebrich M, Sievers HH (2016). Survival and freedom from aortic valve-related reoperation after valve-sparing aortic root replacement in 1015 patients. Interact Cardiovasc Thorac Surg.

[28] Benedetto U, Melina G, Takkenberg JJ, Roscitano A, Angeloni E, Sinatra R (2011). Surgical management of aortic root disease in Marfan syndrome: a systematic review and meta-analysis. Heart.

